# Supplementary material for: The Association between Nursing Skill Mix and Patient Outcomes in a Mental Health Setting: Protocol for an Observational Feasibility Study
Source: Int J Environ Res Public Health. 2022 Apr 5;19(7):4357. doi: 10.3390/ijerph19074357 (PMC8998938; doi:10.3390/ijerph19074357)
Supplement: Supplementary file 1 [file ijerph-19-04357-s001.zip › ijerph-1615697-supplementary.pdf]

International Journal of
Environmental Research and Public Health

MDPI

Table S1. STROBE Statement—Checklist of items that should be included in reports of cohort studies.

Section and Item	Item No	Recommendation	Reported on Page
**Title and abstract**	1	(*a*) Indicate the study's design with a commonly used term in the title or the abstract	1
		(*b*) Provide in the abstract an informative and balanced summary of what was done and what was found	1
**Introduction**			
Background/rationale	2	Explain the scientific background and rationale for the investigation being reported	1 - 2
Objectives	3	State specific objectives, including any prespecified hypotheses	3
**Methods**			
Study design	4	Present key elements of study design early in the paper	3 - 7
Setting	5	Describe the setting, locations, and relevant dates, including periods of recruitment, exposure, follow-up, and data collection	3 - 7
Participants	6	(*a*) Give the eligibility criteria, and the sources and methods of selection of participants. Describe methods of follow-up	3
		(*b*) For matched studies, give matching criteria and number of exposed and unexposed	N/A
Variables	7	Clearly define all outcomes, exposures, predictors, potential confounders, and effect modifiers. Give diagnostic criteria, if applicable	3 - 7
Data sources/ measurement	8	For each variable of interest, give sources of data and details of methods of assessment (measurement). Describe comparability of assessment methods if there is more than one group	4 - 5
Bias	9	Describe any efforts to address potential sources of bias	7
Study size	10	Explain how the study size was arrived at	3
Quantitative variables	11	Explain how quantitative variables were handled in the analyses. If applicable, describe which groupings were chosen and why	6 - 7
Statistical methods	12	(*a*) Describe all statistical methods, including those used to control for confounding	6 - 7
		(*b*) Describe any methods used to examine subgroups and interactions	6 - 7
		(*c*) Explain how missing data were addressed	6 - 7
		(*d*) If applicable, explain how loss to follow-up was addressed	6 - 7
		(*e*) Describe any sensitivity analyses	N/A
**Results**			
Participants	13	(a) Report numbers of individuals at each stage of study—eg numbers potentially eligible, examined for eligibility, confirmed eligible, included in the study, completing follow-up, and analysed	N/A
		(b) Give reasons for non-participation at each stage	N/A
		(c) Consider use of a flow diagram	N/A
Descriptive data	14	(a) Give characteristics of study participants (eg demographic, clinical, social) and information on exposures and potential confounders	N/A
		(b) Indicate number of participants with missing data for each variable of interest	N/A
		(c) Summarise follow-up time (eg, average and total amount)	N/A
Outcome data	15	Report numbers of outcome events or summary measures over time	N/A

Section and Item	Item No	Recommendation	Reported on Page
Main results	16	(*a*) Give unadjusted estimates and, if applicable, confounder-adjusted estimates and their precision (eg, 95% confidence interval). Make clear which confounders were adjusted for and why they were included	8
		(*b*) Report category boundaries when continuous variables were categorized	N/A
		(*c*) If relevant, consider translating estimates of relative risk into absolute risk for a meaningful time period	N/A
Other analyses	17	Report other analyses done—eg analyses of subgroups and interactions, and sensitivity analyses	N/A
**Discussion**			
Key results	18	Summarise key results with reference to study objectives	N/A
Limitations	19	Discuss limitations of the study, taking into account sources of potential bias or imprecision. Discuss both direction and magnitude of any potential bias	8
Interpretation	20	Give a cautious overall interpretation of results considering objectives, limitations, multiplicity of analyses, results from similar studies, and other relevant evidence	N/A
Generalisability	21	Discuss the generalisability (external validity) of the study results	N/A
**Other information**			
Funding	22	Give the source of funding and the role of the funders for the present study and, if applicable, for the original study on which the present article is based	8

Table S2. Data extraction form for patients

Patient study identification number	Age (record the patient's age in years at point of hospital admission)	Gender (code: 1. Male, 2. Female, 3. Other)	What was the patient's employment status at the point of admission: (Code: 1. Employed, 2. Unemployed, 3. Student, 4. Retired, 5. Other)	What was the patient's primary psychiatric diagnosis? (Code: 1. Mood and/or anxiety disorder [including bipolar], 2. Schizophrenia, 3. Personality disorders, 4. Other [specify])	Were any psychiatric comorbidities recorded? (Code: 1. Yes, 2. No)	Was any substance use recorded? (Code: 1. Yes, 2. No)	Were any physical comorbidities recorded? (Code: 1. Yes, 2. No)	When was the patient admitted to the ward? (xx/xx/20xx)	Was the patient admitted under mental health law (Code: 1. Yes, 2. No)	Was the patient on Community Treatment Order prior to admission to hospital? (Code: 1. Yes, 2. No)	When was the patient discharged from the participating ward? (xx/xx/20xx)	Was the patient discharged on a Community Treatment Order? (Code: 1. Yes, 2. No)	Was the patient readmitted to inpatient psychiatric services at the participating health service within 12 months of the follow-up period (Code: 1. Yes, 2. No)	When was the patient readmitted to inpatient psychiatric services at the participating health service? (dd/mm/20yy).	How many readmissions to the psychiatric ward did the patient have during the study period? (Recorded as number)	HoNOS (Health of the national Outcome Scale) total score on admission to hospital (recorded as number)	HoNOS (Health of the national Outcome Scale) total score immediately prior to discharge (recorded as number)
1																	
2																	
3																	
4																	

Note. The study period is from 6th January 2020 to 5th March 2021 (The time frame includes 60 days of inpatient admission and 12 months of follow-up period after hospital discharge). Note that four patients are for illustrative purposes only.

Table S3. Nurse roster data extraction form (week 1)

Day/date	How many nurses in each group were working each day? A day is considered to be the Morning (7:00am though 3pm), Afternoon (1pm though 9pm) and Night shift (9pm though 6:59 am) added together.						Total number of nurses for each day	Total number of inpatients at midnight for each day
	Mental Health nurses	Comprehensive nurses	Enrolled Nurses	Nursing Assistants	Agency nurses	Student nurses		
Monday 06/01/2020								
Tuesday 07/01/2020								
Wednesday 08/01/2020								
Thursday 09/01/2020								
Friday 10/01/2020								
Saturday 11/01/2020								
Sunday 12/01/2020								

Note. Roster data to be extracted from the 6th of January 2020 through 12th January 2020 inclusive. Seven days of data extractions are shown for illustrative purposes only.
